# Constructing and Communicating COVID-19 Stigma on Twitter: A Content Analysis of Tweets during the Early Stage of the COVID-19 Outbreak

**DOI:** 10.3390/ijerph17186847

**Published:** 2020-09-19

**Authors:** Yachao Li, Sylvia Twersky, Kelsey Ignace, Mei Zhao, Radhika Purandare, Breeda Bennett-Jones, Scott R. Weaver

**Affiliations:** 1 Department of Communication Studies, The College of New Jersey, Ewing, NJ 08628, USA; purandr2@tcnj.edu (R.P.); bennett4@tcnj.edu (B.B.-J.); 2 Department of Public Health, The College of New Jersey, Ewing, NJ 08628, USA; twerskys@tcnj.edu (S.T.); ignacek1@tcnj.edu (K.I.); zhaom@tcnj.edu (M.Z.); 3 School of Public Health, Georgia State University, Atlanta, GA 30303, USA; srweaver@gsu.edu

**Keywords:** COVID-19, coronavirus 2019, Twitter, stigma, model of stigma communication, content analysis

## Abstract

This study focuses on stigma communication about COVID-19 on Twitter in the early stage of the outbreak, given the lack of information and rapid global expansion of new cases during this period. Guided by the model of stigma communication, we examine four types of message content, namely mark, group labeling, responsibility, and peril, that are instrumental in forming stigma beliefs and sharing stigma messages. We also explore whether the presence of misinformation and conspiracy theories in COVID-19-related tweets is associated with the presence of COVID-19 stigma content. A total of 155,353 unique COVID-19-related tweets posted between December 31, 2019, and March 13, 2020, were identified, from which 7000 tweets were randomly selected for manual coding. Results showed that the peril of COVID-19 was mentioned the most often, followed by mark, responsibility, and group labeling content. Tweets with conspiracy theories were more likely to include group labeling and responsibility information, but less likely to mention COVID-19 peril. Public health agencies should be aware of the unintentional stigmatization of COVID-19 in public health messages and the urgency to engage and educate the public about the facts of COVID-19.

## 1. Introduction

On December 31, 2019, Wuhan Municipal Health Commission reported a cluster of cases of pneumonia in Wuhan, China, and shortly thereafter the novel coronavirus (SARS-CoV-2) came to the public’s attention. On February 11, 2020, the World Health Organization (WHO) officially named the disease resulting from infection by the novel coronavirus “coronavirus disease 2019,” abbreviated as COVID-19 [1]. COVID-19 is a recently identified infectious disease that can lead to severe health consequences [2,3,4]. Deeply concerned by the “alarming levels of spread and severity,” WHO characterized COVID-19 as a pandemic on March 11, 2020 [1]. As this public health emergency evolves, community members find themselves increasingly frightened and concerned by the many uncertainties regarding the novel disease and the surging number of cases and deaths across the world [5,6]. The heightened fear and anxiety about COVID-19 and the tendency to associate negative emotions with outgroup members can lead to COVID-19 stigma, which involves negative attitudes and discrimination against people with characteristics that are perceived to make them more likely to contract and spread COVID-19 [5].

During infectious disease outbreaks, individuals are recommended to comply with specific guidelines to prevent infection and reduce further disease transmission, such as self-quarantining, seeking medical treatment, and reporting contacts to public health officials. However, stigma can deter people from adopting these behaviors [7]. For instance, HIV-related stigma has been adversely associated with HIV disclosure [8] and medication adherence [9], which are crucial to HIV treatment and prevention. Higher HIV-related stigma is also related to increased depression, substance abuse, and sexual risk-taking [10,11,12]. Similarly, higher tuberculosis-related stigma has been related to lower levels of testing and treatment seeking, as well as difficulties with contact tracing during tuberculosis outbreaks [13,14]. Given that COVID-19 is infectious, contact tracing is vital to assess community spread. Yet, COVID-19 stigma could prevent people from seeking COVID-19 testing, and if they test positive, from disclosing their contacts. COVID-19 stigma can also deter people from adopting certain precautions, such as wearing face masks, and seeking proper health care. Therefore, reducing COVID-19 stigma is crucial in order to control the spread of COVID-19.

Stigma is constructed and communicated via social interactions. The model of stigma communication [15,16] states that four types of message content, namely mark, group labeling, responsibility, and peril, are instrumental in forming stigma beliefs, motivating people to share stigma messages, and encouraging people to discriminate against stigmatized groups. Marks are cues used to identify members of a stigmatized group, such as malformed limbs for people with a physical disability and open sores for people with certain infectious diseases [16]. In the COVID-19 context, having flu-like symptoms, wearing a face mask, or being Asian may be perceived as marks for COVID-19 stigma. Labels are descriptions of the stigmatized group as a separate group entity. For instance, labeling someone as “HIV positive” or “having AIDS” triggers stigmatization for people living with HIV [17]. Likewise, when COVID-19 is labeled as the Chinese virus or Wuhan virus, stigma is created towards Chinese people and Wuhan residents [6]. Given the tendency to lump and view other racial groups as heterogenous, the label may also be generalized to all Asian individuals. Responsibility is information in messages that implies blame by making attributions about a person’s choice and control. For instance, men who have sex with men are often accused of choosing their “lifestyle” and thus, should be responsible for being infected and transmitting HIV [18]. In the context of COVID-19, international travelers may be perceived as choosing to travel and therefore, should take responsibility for the outbreak. Peril is information that links the marked, labeled, responsible individuals to physical or social danger that threatens the well-being of the community. Infectious diseases are particularly threatening to group functioning due to the sickness and death of group members [16]. Thus, when people discuss the health and social impact of COVID-19, they may intentionally or unintentionally create stigma towards people diagnosed with COVID-19 or those perceived to have COVID-19. Taken together, messages with the content of mark, group labeling, responsibility, and peril are likely to construct and spread COVID-19 stigma.

Twitter is a global online social media platform where users produce and share short posts called tweets. There are currently 166 million daily Twitter users who create nearly 500 million tweets each day [19]. Since the beginning of the COVID-19 outbreak, Twitter has become a crucial platform for the general public to seek and share information, form perceptions, and express opinions about COVID-19 [6,20,21,22]. To better understand COVID-19 stigma on Twitter, we explore the message content of stigma communication. Specifically, guided by the model of stigma communication, our first study goal is to identify specific themes of the mark, label, responsibility, and peril content of COVID-19 related tweets during the beginning of the outbreak. The results would provide us with a more nuanced understanding of public perceptions of COVID-19 on Twitter and how COVID-19 stigma is created and communicated via social interactions on social media.

In addition, misinformation and conspiracy theories of COVID-19 may also facilitate COVID-19 stigma given that they can contribute to the mark, group labeling, responsibility, and peril of the disease [5]. For instance, the false perception that Asian people are more likely to have and transmit COVID-19 makes Asian physical features a mark for COVID-19. The unsubstantiated idea that COVID-19 is a bioweapon created by the Chinese government may encourage people to label the disease as a “Chinese virus”, attribute responsibility for the harms of COVID-19 to Chinese people and amplify the peril of COVID-19. Thus, our second study objective is to examine whether the presence of misinformation and conspiracy theories in COVID-19 related tweets is associated with the presence of COVID-19 stigma content. The results would add to prior studies examining the content of misinformation and conspiracy theories of COVID-19 [21,23], help us better understand how misinformation and conspiracy theories are related to COVID-19 stigma, and provide means to reduce COVID-19 stigma.

## 2. Materials and Methods

### 2.1. Twitter Data

We utilized the statistical program R rtweet package [24] and a developer application programming interface (API) to retrieve English language tweets posted between 31 December 2019, when Chinese officials first reported the novel pneumonia cases, and 13 March 2020. A simple random sample of 500 tweets per day were collected from 31 December 2019 through 25 February 2020. We randomly sampled 18,000 tweets per day from 26 February to 13 March 2020, oversampling given the rapid global expansion of COVID-19 cases during this period. The key words used for the search were #Wuhan, Wuhan, Coronavirus, #Coronavirus, and COVID.

To reduce the influences of tweet bots (i.e., automated accounts that use software to post content and even interact with other users and are often used to spread misinformation or alter social discourse) that may constitute up to 45% of tweets about COVID-19 [25], sources for the tweets were restricted to those that were more likely to be individuals tweeting, such as Twitter for iPhone, Twitter for Android, and Twitter Web App. Duplicates and retweets were also excluded, resulting in 155,353 unique tweets. A simple random sample of 7000 tweets were selected for manual coding and data analysis.

### 2.2. Coding Scheme

The coding team consisted of four members, who received a minimum of four 2-h training sessions. Each coder was randomly assigned 2000 tweets, including 1000 unique tweets and 1000 overlap tweets with the other coders. Coders read their assigned tweets multiple times to be familiar with the data. Then, based on the model of stigma communication, coders independently generated a list of topics related to mark, group labeling, responsibility, and peril of COVID-19. The coders then compared the degree of overlap between their lists. Finally, a codebook was developed, in which the four types of stigma message content are overarching themes and the specific topics identified in the open coding process are coding variables under those themes. The codebook also includes a misinformation variable and a conspiracy theory variable. Table 1 specifies all the coding variables.

### 2.3. Content Analysis

A content analysis was conducted on a random sample of 7000 tweets to identify the presence of the coding constructs (0 = absent, 1 = present). The unit of analysis was each individual tweet. Table 1 presents the percentage of times the variables were identified in the dataset and examples of the variables. For each variable, the coding categories were mutually exclusive; that is, a variable was either present or absent in a tweet. However, a tweet may include multiple constructs. For instance, in the tweet, “Screw political correctness I’m still calling it the Wuhan virus fucking dog/cat eating fuckers,” the variable of group labeling (“Wuhan virus”) and lifestyle (“fucking dog/cat eating fuckers”) were present. All the four coders independently coded a random 15% of the sample for reliability purposes. Intercoder reliability was high, with Krippendorff’s alphas ranging from 0.75 to 0.96 (refer to Table 1 for intercoder reliability for each variable). The remaining tweets were divided evenly and randomly assigned to each coder.

## 3. Results

### 3.1. Specific Topics of COVID-19 Stigma

Our first study goal is to understand the specific topics of mark, group labeling, responsibility, and peril in COVID-19 related tweets. Approximately 25% of the 7000 tweets (1759) included at least one type of message content that is instrumental in developing and disseminating COVID-19 stigma. Specifically, 22.56%, 2.51%, and 0.06% of the 7000 tweets included one, two, and three types of stigma message content, respectively. No tweets included all the four components.

#### 3.1.1. Mark

Marks are cues to identify members of a stigmatized group. Among the 243 tweets (3.47%) that included marks for COVID-19, four specific types of marks emerged from the data, including flu-like symptoms, personal protective equipment (PPE), Asian origin, and healthcare providers and essential workers. About 1.44% of tweets mentioned that because a person has flu-like symptoms, they may have or transmit COVID-19. For instance, a user posted, “I have a terrible coughing spell at work and these people probably think I have coronavirus. Whole time I’m choking on a piece of lint.” Another user shared a story where a family was denied service due to a daughter coughing, “Panicked passengers get family kicked off flight over coughing daughter…” Here, people use flu-like symptoms to identify people who (may) have COVID-19. In addition, 1.67% of tweets mentioned that people may have COVID-19 if they are using PPE, such as face masks, goggles, and shoe covers. A user wrote, “If they are healthy, why are they wearing face masks and goggles?” Another posted, “Face mask is for those with suspected signs. Leave protective gears for health workers and affected persons.” By linking PPE with “suspected signs,” people turned PPE into a mark to single out people with COVID-19.

Because COVID-19 was first reported in China, another mark for COVID-19 emerged in the data was Asian origin. About 2.11% of tweets mentioned that because a person is Asian, a product is made in Asia, or a place is linked to Asia (e.g., Chinatown, Chinese restaurant), that person, product, or place may have or transmit COVID-19. A user wrote, “I ordered a package from China before this coronavirus stuff became serious and once it gets here, I’m spraying it with alcohol, tying it up in a bag and throwing it in the garage.” Similarly, another user suggested that face masks from China may also have COVID-19, “When you put on a mask to avoid coronavirus but realized mask has also been made in china.” About 0.83% of tweets also mentioned that Asian people have “coronavirus privilege,” meaning that they are more likely to contract and spread COVID-19. Those statements linked Asian descent to COVID-19. Moreover, a few (0.17%) tweets also considered certain careers indicators for having or transmitting COVID-19, including healthcare providers (e.g., “I need to stay away from my nurse neighbor. She may make me sick.”) or essential workers (e.g., “My sister has to self-isolated herself since she works at a grocery store and my parents think she will get them sick.”). In short, flu-like symptoms, PPE, Asian origin, and certain careers are used to mark people who may have and spread COVID-19.

#### 3.1.2. Group Labeling

In stigma communication, labels are created to categorize the stigmatized group as a separate group entity. A total of 83 tweets (1.19%) included group labeling and two types of labels emerged from the data. About 0.86% of tweets referred to COVID-19 as “Wuhan virus,” “Chinese/China virus,” or “Asian virus,” separating Wuhan residents, Chinese people, or Asian individuals from the general population and suggesting that those groups are more susceptible to COVID-19 and are threats to other communities. Some tweets explicitly defended such stigmatized names as “accurate” and “factual,” because they specify the origin of the disease. For instance, a user wrote, “#COVID-19 originated from #wuhan to call it #wuhanvirus is a factual statement.” Another said, “Actually calling it coronavirus is super generalized because SARS and MERS were types of coronaviruses and calling it Wuhan coronavirus or China virus actually gives it a specification based on where it originated like MERS name does.”

A few tweets (0.33%) also referred to COVID-19 as “trumpvirus” or “trumpdemic.” A user wrote, “the #coronavirus does not care if you’re a republican. This pandemic of #covid19 is not a false story. So remain calm and do not blame the dem’s for the #trumpdemic.” Another user posted, “@xxx so you think #coronavirus is a #hoax? #trumpvirus.” While users often utilized the terms to express their political views, labeling a disease with the name of a controversial political leader may unintentionally impose negativity on people who have COVID-19, facilitating the stigmatization of COVID-19.

#### 3.1.3. Responsibility

Responsibility is message content that implies blame by making attributions about individual’s choices and control. These messages blame people for purposefully engaging in certain behaviors that may put them in the stigmatized situations, in this case as a vector for COVID-19. In total, 124 tweets (1.77%) included responsibility information and we identified three specific types of behaviors using blaming language. First, about 0.30% of tweets blamed COVID-19 on people who have different culturally linked food preferences. One user wrote, “um guys we shouldn’t support BTS [author note: A South Korean boy band] because those sick ass Chinese people trying to have a concert in the U.S. and give us the coronavirus. It’s just rude and disgusting us whites don’t want to get sick from them bat eating fuckers.” Another tweet read, “All this coronavirus shit all because someone ate something weird. Well I hope their dead.” Here, the tweets implied that people who have different eating habits should be held responsible for COVID-19. This aspect of stigma creates a cultural responsibility for the COVID-19 outbreak due to food preferences and stigmatizes all Asian populations.

Travelers were also being blamed for contracting and spreading COVID-19 in approximately 0.91% tweets. A user wrote, “People from abroad who have the coronavirus and have paid for a trip to UK are still going to travel here because they can’t get their money back.” Another posted, “It blows my mind the number of people still willing to travel with this COVID-19. it’s going to be interesting when the snowbirds get home.” Those tweets suggested that because people purposely chose to travel, they should be held accountable for the surging cases of COVID-19. Similarly, about 0.59% of tweets also blamed people who choose not to adopt COVID-19 precautions. A tweet read, “You are stupid and ignorant if you still don’t take the coronavirus situation seriously. wear a mask. wash your hands frequently. take responsibility of your life.” Another user wrote, “What I don’t understand is ppl [people] who put their mouth on the dome lids for slushies; then touch it back to another flavor nozzle... triflin! I just watched a video of a girl on sc [Snapchat]; no one thought twice about it; u [you] guys wonder why ppl have the coronavirus bc yall [because you’re all] disgusting humans.” Here, the tweets implied that people who do not follow precautions “deserved COVID-19” because they choose not to comply with public health guidelines, which put themselves and other community members in danger.

#### 3.1.4. Peril

Peril is message content underlining the danger that a stigmatized group poses to the rest of the society. In the context of infectious diseases, threats of the diseases are common peril information that evoke and amplify stigma related to the diseases [16]. About one in five tweets (1396, 19.94%) mentioned threats of COVID-19 on people’s health, their normal life, the economy, and healthcare systems.

About 9.34% of tweets mentioned the negative mental and physical health consequences of COVID-19. Some users highlighted high severity and susceptibility of COVID-19: “It is way worse than flu,” “Has higher death rates than flu,” “It’s about the rate of mortality of the coronavirus which is what makes it more dangerous and also how infectious it is,” and “About 70% of the world population will get it.” Other tweets mentioned the adverse mental health impact of COVID-19. One tweet read, “Work is so stressful. Come home news is so stressful. And I’m living in fear of bringing home coronavirus!!!” Another user wrote, “I’m so stressed about COVID-19 and today is our travel day out of TX through Atlanta to Newark. They say the body keeps the score and as much as I try to remain calm, I’ve triggered a period even though I have an IUD [intrauterine contraceptive device]. This is great.” These examples demonstrate Twitter users’ strong concerns about the health threats of COVID-19.

Nearly 6.84% of tweets focused on how COVID-19 threatens people’s ability to live a normal life, such as lack of daily resources (e.g., “Went to CVS to get some Dayquil and they’re cleaned out. I mean the shelves were empty. Is that what people are stocking up on for coronavirus??”), cancellations of vacations or activities (e.g., “Stupid coronavirus! Ruined my entire vacation plan! Today, at this time, I would’ve been chilling with @xxx in Singapore”), and home schooling (e.g., “They will close campus after Spring break. Welcome to Zoom University at my bedroom!”). These tweets reflect the concern about disruptions in daily life due to COVID-19.

Moreover, 3.93% of tweets highlighted the adverse impact of COVID-19 on the economy, such as unemployment, pay cuts, decreased GDP, and crashed stock market. For example, “If revenue continues to fall as a consequence of the coronavirus, firms will make employees redundant to cut costs,” “The first U.S. layoffs from the coronavirus are here — with more feared to come,” and “I’m feeling sick and it is not because of COVID-19 it’s the stock market ride.” Those examples indicate that in the beginning of the crisis, the economic peril of COVID-19 was on people’s minds.

A few users (1.13%) also worried about the burden of COVID-19 on the healthcare system. One user wrote, “This new normal is going to be hard and scary. but we have to start now. before we think we need to. Hospitals won’t be able to handle the massive influx.” Another posted, “If this coronavirus is so contagious, then our healthcare staff are also highly susceptible to also being struck down with the virus. who is going to man the hospitals in this case? Very concerning.” These examples show that COVID-19 poses danger to society by threatening healthcare systems.

About one in five tweets highlighted the peril of COVID-19 in various aspects of people’s life. While these are legitimate concerns related to COVID-19 and most of the information may not intend to stigmatize people with COVID-19, the fear and anxiety associated with the threats of COVID-19 can encourage people to mark, label, and blame “others” for the situation, which facilitates the creation and spread of COVID-19 stigma.

### 3.2. Misinformation, Conspiracy Theories, and COVID-19 Stigma

Our second study objective is to explore how the presence of COVID-19 misinformation and conspiracy theories is related to the presence of mark, group labeling, responsibility, and peril content in tweets. We coded for the presence of common misinformation about COVID-19 identified in existing studies [26,27], such as COVID-19 “is fake,” “is just a flu,” “heat kills the virus,” “drinking tea will stop the coronavirus,” as well as factually false statements, such as “only Asians will get corona” and “young people will not die from the virus.” When necessary, government sources were used for fact checking. We also coded for the presence of major conspiracy theories of COVID-19 [23,28], such as, “It originated in a lab in Wuhan and some idiots let it loose,” “God sent coronavirus to destroy LGBTQ people,” and “Bill Gates created the virus to test 5G.” In total, 4.21% of the tweets included misinformation about COVID-19 and 2.00% of tweets mentioned at least one COVID-19 conspiracy theories.

Chi-square tests (see Table 2) showed that tweets with misinformation did not include more mark content than those without misinformation, ${Χ}^{2}\;$(1, *N* = 7000) = 2.91, *p* = 0.089. There was no difference in the presence of group labeling between tweets with misinformation and without misinformation, ${Χ}^{2}\;$(1, *N* = 7000) = 3.71, *p* = 0.054. Tweets with misinformation did not include more responsibility information than those without misinformation, ${Χ}^{2}\;$(1, *N* = 7000) = 0.12, *p* = 0.727. Peril of COVID-19 was more likely to be present in tweets without misinformation (20.34%) than those with misinformation (10.85%), ${Χ}^{2}\;$(1, *N* = 7000) = 15.96, *p* < 0.001.

Tweets with conspiracy theories did not mention more mark content than those without conspiracy theories, ${Χ}^{2}\;$(1, *N* = 7000) = 3.72, *p* = 0.054. Group labeling was more likely to be present in tweets with conspiracy theories (6.43%) than those without conspiracy theories (1.08%), ${Χ}^{2}\;$(1, *N* = 7000) = 33.52, *p* < 0.001. Tweets with conspiracy theories (7.14%) were more likely to have responsibility information than those without conspiracy theories (1.66%), ${Χ}^{2}\;$(1, *N* = 7000) = 23.69, *p* < 0.001. Peril of COVID-19 was more likely to be present in tweets without conspiracy theories (20.22%) than those with conspiracy theories (6.43%), ${Χ}^{2}\;$(1, *N* = 7000) = 16.34, *p* < 0.001.

In summary, compared to tweets without misinformation, those with misinformation were less likely to mention the threats of COVID-19. Compared to tweets without conspiracy theories, tweets with conspiracy theories were more likely include group labeling and responsibility information, but less likely to mention the peril of COVID-19.

## 4. Discussion

Twitter has been increasingly used in crisis risk communication because of its capability to reach and engage a wide audience and to exchange information in a timely manner. Thus, an investigation of COVID-19 tweets helps us better understand public opinions about the ever-changing crisis. Guided by the model of stigma communication, we examined how twitter users intentionally and unintentionally facilitate the creation and dissemination of COVID-19 stigma by tweeting four types of stigma message content. Results showed that the peril of COVID-19 was mentioned the most often, followed by mark, group labeling, and responsibility. In addition, tweets with misinformation and/or conspiracy theories were less likely to include peril information than tweets without misinformation and/or conspiracy theories. Conspiracy theory tweets were more likely to label COVID-19 as “Wuhan/Chinese virus” and to blame others for the outbreak. Overall, this study provides a snapshot of COVID-19 stigma communication in the beginning of the pandemic, as well as offers practical implications for public health agencies to reduce COVID-19 stigma.

### 4.1. Practical Implications

One of the controversies around COVID-19 protective measures in the United States is about mask wearing. Our study shows that, in the beginning of the epidemic, mask wearing was considered a mark for COVID-19. This may play a role in the reluctance of the public to adopt precautionary mask wearing to prevent the spread of the disease. The WHO initially suggested that, in the general community, only people who are symptomatic or who are caring for people with COVID-19 should wear masks. In April 2020, the WHO stated, “the wide use of masks by healthy people in the community setting is not supported by current evidence and carries uncertainties and critical risks” [29]. Those messages may unintentionally contribute to COVID-19 stigma by linking prevention measures like mask wearing with disease infection and transmission. Thus, public health messages should minimize the unintentional stigmatization of infectious diseases by emphasizing the effectiveness of prevention measures, rather than associating precautions to certain groups of people.

Historically, infectious diseases were often named after its perceived origin, such as the “Spanish flu” and “Rift Valley Fever.” However, attaching locations to the disease can mislead people to focus on the disease’s past origins rather than its present threat, contributing to a lack of prevention actions in the public. Moreover, linking an infectious disease to certain locations or ethnicities creates stigma towards people from those places or in those ethnic groups [5,15]. Due to the initial widespread use of “Wuhan/China Virus” and the lack of official name from the WHO in the first month of the crisis, anti-Asian bias and hate crimes worldwide have surged [30,31]. In addition, some tweets attributed the origin of COVID-19 to culturally Chinese foods. This is not a new phenomenon as previous political and media discourse have blamed the plague in the early 1900′s and the 2003 severe acute respiratory syndrome (SARS) outbreak on Chinese culture and eating habits [32]. Given that experiences of disease-related stigma are directly linked to adverse mental health outcomes [33,34], we advocate greater efforts to reduce discrimination against Asian people and a timelier rollout of a non-stigmatizing name of infectious diseases to avoid widespread stigmatization.

Among the four types of stigma message content, peril was mentioned the most frequently. Twitter users underlined the threats that COVID-19 poses to their health, people’s normal life, the economy, and the healthcare system. Those concerns and the associated anxiety and fear contribute to COVID-19 stigma. Notably, when misinformation was present in a tweet, the tweet was less likely to include peril information. This may be because the misinformation often downplayed the severity of COVID-19 or even denied the existence of the disease. In addition, compared to tweets without conspiracy theories, tweets with conspiracy theories were more likely to attach locations or ethnicity to the disease (labeling), more likely to blame others for the situation (responsibility), but less likely to indicate the threat of COVID-19 (peril). The results support that misinformation and conspiracy theories facilitate the creation and dissemination of COVID-19 stigma. Thus, another way to reduce the stigma is to specifically address conspiracy theories, engage critical thinking skills, and use effective communication strategies to spread the facts. The WHO and other world organizations have suggested using simple language and social media to engage and educate the general public about the facts of COVID-19 [5].

### 4.2. Limitations and Future Research

Although nearly 25% of the 7000 tweets included at least one type of stigma message content, about three-fourths (76.35%) of those tweets only mentioned the peril of COVID-19 without mark, group labeling, or responsibility content. Peril may be an instrumental component of stigma communication, but peril alone may not be sufficient to create and communicate stigma. In other words, tweets with only peril information may not intend to stigmatize people with COVID-19. However, tweets are not received in isolation of one another or messages communicated via other channels. Tweets conveying peril, when consumed with other tweets that marks or labels, may facilitate stigma formation and sharing. Moreover, even if about 6% of tweets feature stigma communication, the messages can still reach a larger audience via user exposure and engagement. Future studies should investigate the amplification of stigma messages by considering the tweets’ exposure in terms of the number of followers of the Twitter accounts and the number of likes, comments, and retweets of tweets perpetuating stigma communication. In addition, future studies should explore whether tweets with certain stigma message content are more likely to be liked, commented, and retweeted. Future research should also examine whether results of this study generalize to other social media platforms.

Another limitation is that English language tweets did not generally have an associated a geocode and; therefore, could be posted by users from any country, between which the rates of COVID-19, precaution measures, and public health communication may be different. In addition, we randomly selected and examined 7000 tweets. While the number of tweets being analyzed is large based on the capability of human coding, future research should utilize assisted coding methods, such as supervised machine learning, to examine a larger sample. Epidemiology and our understanding of COVID-19 has also been changing over time. This study captures a small window in the very beginning of the outbreak as it grew to become a global pandemic and thus, reflects attitudes and knowledge from that time period only. Future research should track potential changes in stigma communication and misinformation propagated over time to investigate if, as people became more knowledgeable about COVID-19 or as COVID-19 rates changed, stigma communication changes. Moreover, videos of individuals who refused to wear masks have been recently circulated on social and mainstream media. Another future direction is to explore what those videos reveal about stigma and misinformation about COVID-19 and attitudes towards primary prevention strategies.

## 5. Conclusions

In the beginning of the COVID-19 crisis, about 3.47%, 1.19%, 1.77%, and 19.94% of tweets in this study mentioned mark, group labeling, responsibility, and peril, respectively, which are instrumental in developing and disseminating COVID-19 stigma. Stigma message content was also more likely to appear in tweets that contained misinformation and conspiracy theories. Given that COVID-19 stigma can diminish the efforts to combat the disease and result in adverse health consequences for stigmatized populations, public health agencies should be aware of the unintentional stigmatization of COVID-19 in public health messages and the urgency to engage and educate the public about the facts of COVID-19.

## Figures and Tables

**Table 1:** Examples, intercoder reliability, and percentages of coding variables.

Coding Variables	Examples	Intercoder Reliability	Percentages (*N* = 7000)
**Mark**			3.47%
1. Flu-like symptoms	“The more I read about the coronavirus, the more I freak out when I hear someone cough or sneeze in public.”	0.81	1.44%
2. Personal protective equipment	“A Chinese girl was kicked off my train because she was wearing a facemask.”	0.81	1.67%
3. Asian Origin	“Asians should be banned from the US due to their ‘coronavirus privilege.’”	0.82	2.11%
4. Healthcare providers and essential workers	“I had a customer complained today bc he didn’t want to sit next to a nurse as they would get him sick.”	0.77	0.17%
**Group Labeling**			1.19%
1. Wuhan/China/Asian virus	“Actually, I prefer calling it Wuhan virus, more fitting.”	0.90	0.86%
2. Trump virus	“Yes #trumpvirus is additionally deadly because he is in the WH [White House]. But don’t forget you are endangered because the GOP allow his actions.”	0.91	0.33%
**Responsibility**			1.77%
1. Different eating habits	“@xxx you can’t blame anyone but the restaurant [who served] and the people who ate bat soup in Wuhan, China.”	0.88	0.30%
2. Travelling	“Coronavirus is running rampant in Europe right now and all these white girls on Instagram still be taking vacations to the beach.”	0.89	0.91%
3. Violating precautions	“@xxx this is so irresponsible. ‘India is magical so coronavirus can’t infect us!’ ?! No data supports this theory. COVID-19 is not the flu. Failure to take the threat seriously and delaying social distancing practices will result in faster spread.”	0.75	0.59%
**Peril**			19.94%
1. Health	“The #coronaoutbreak will kill many people and temporarily disable others.”	0.84	9.34%
2. Normal life	“Oh my god I just watched the news on tv the babies the coronavirus is progressing at my place they are making the decision to close all the schools and the public for 14 days it’s really scary I’m scared for my family and my mom.”	0.84	6.84%
3. Economy	“Things coronavirus will affect: house prices will crash, stock markets crash, unemployment will increase.”	0.85	3.93%
4. Healthcare system	“In a way the relatively low mortality rate makes the #coronavirus more of a problem as it spreads more widely and causes more strain on hospitals.”	0.85	1.13%
**Misinformation**	“7 million Americans get the flu annually and 61,000 die yet we have a vaccine. only 496 cases of Wuhan virus and 23 deaths. you, anti-trump celebs, the media, and Dems are weaponizing this for political gain and it’s not just irresponsible it’s criminal.”	0.96	4.21%
**Conspiracy Theories**	“The aliens are coming they started the coronavirus they are trying to kill us.”	0.94	2.00%

**Table 2:** Percentages of stigma communication message content in tweets with and without misinformation and conspiracy theories.

Stigma Message Content	Misinformation		Conspiracy Theories	
	Absent (*n* = 6705, %)	Present (*n* = 295, %)	Absent (*n* = 6860, %)	Present (*n* = 140, %)
Mark	3.54_a_	1.69_a_	3.41_a_	6.43_a_
Group Labeling	1.13_a_	2.37_a_	1.08_a_	6.43_b_
Responsibility	1.76_a_	2.03_a_	1.66_a_	7.14_b_
Peril	20.34_b_	10.85_a_	20.22_b_	6.43_a_

Note: Percentages in every two columns are within condition percentages. Percentages that do not share a subscript letter differ at *p* < 0.05 by the post-hoc multi-group comparison using the Bonferroni correction.
